# Supplementary material for: Enhancer-associated H3K4 methylation safeguards in vitro germline competence
Source: Nat Commun. 2021 Oct 1;12:5771. doi: 10.1038/s41467-021-26065-6 (PMC8486853; doi:10.1038/s41467-021-26065-6)
Supplement: Supplementary file 1 — Supplementary Information [file 41467_2021_26065_MOESM1_ESM.pdf]

## Supplemental Information

# Enhancer-associated H3K4 methylation safeguards *in vitro* germline competence

Tore Bleckwehl, Giuliano Crispatzu, Kaitlin Schaaf, Patricia Respuela, Michaela Bartusel, Laura Benson, Stephen J. Clark, Kristel M. Dorighi, Antonio Barral, Magdalena Laugsch, Wilfred F. J. van IJcken, Miguel Manzanares, Joanna Wysocka, Wolf Reik, Álvaro Rada-Iglesias

# Supplementary Fig. 1

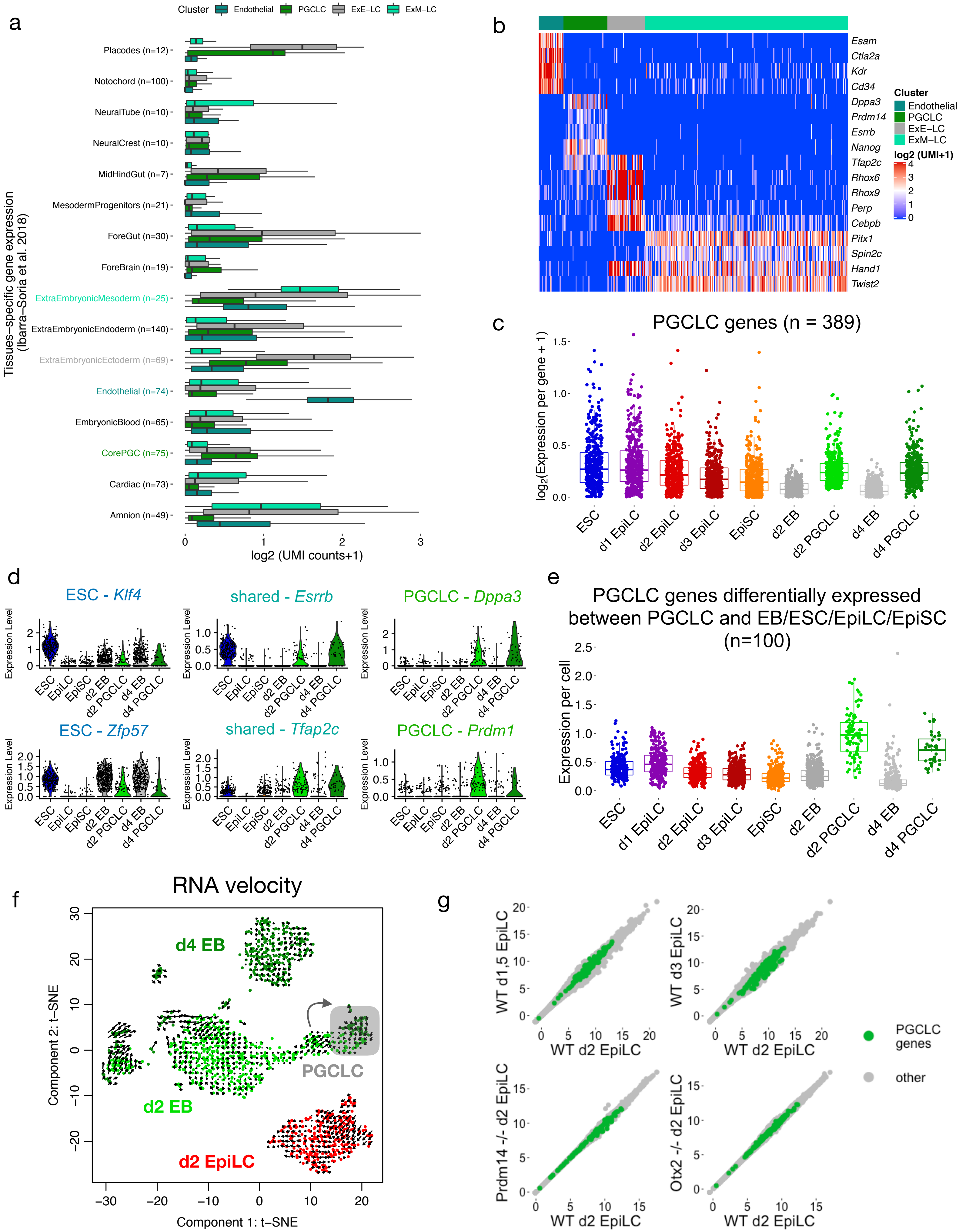

**Supplementary Fig.1: Characterization of the PGCLC differentiation system by scRNA-seq (related to Fig. 1).**

a.) The average gene expression for the specific markers defining the main embryonic and extraembryonic tissues found within E8.25 mouse embryos[1] is shown for the cell clusters identified within d4 EBs. Since PGC markers were not identified by Ibarra-Soria *et al.* 2018[1], we used the core PGC gene set previously defined by Nakaki *et al*. 2016[2] as a representative set of PGC markers expressed in E9.5 embryos. The numbers next to each tissue name indicate the sample size considered for each tissue (tissue markers with logFC >2.5). The horizontal lines in the boxplots indicate the median, the boxes the first and third quartiles and the whiskers the ± 1.5 × interquartile range.

b.) Heatmap showing the expression of representative markers for the main four cell clusters identified within d4 EBs (Fig. 1C; Endothelial, PGCLC, ExE-LC and ExM-LC). The expression values are displayed as UMI (unique molecular identifier) counts in log2 scale.

c.) Expression dynamics of the PGCLC genes during PGCLC formation. Each dot represents the mean expression of a PGCLC gene in all cells belonging to the indicated stages. EB refers to any cell of the EB except those identified as PGCLC (n = 389). The horizontal lines in the boxplots indicate the median, the boxes the first and third quartiles and the whiskers the ± 1.5 × interquartile range.

d.) Violin plots showing the expression of selected ESC markers (*Klf4, Zfp57*), genes active in both ESC and PGCLC (shared: *Esrrb, Tfap2c*), and PGCLC markers (*Dppa3, Prdm1*) in 2i ESC, d2 EpiLC, EpiSC, d2 EB, d4 EB and the PGCLC cluster. EB refers to all cells of the EBs, except those found within the PGCLC cluster.

e.) PGCLC genes specifically expressed in PGCLC were identified by performing a differential expression analysis of PGCLC *vs* EB/ESC/EpiLC/EpiSC. The expression dynamics of the resulting PGCLC-specific genes (n=100) is shown, with each dot representing the mean expression of the PGCLC-specific genes in a single cell belonging to the indicated stages. EB refers to all cells of the EB except those found within the PGCLC cluster. The horizontal lines in the boxplots indicate the median, the boxes the first and third quartiles and the whiskers the ± 1.5 × interquartile range.

f.) RNA velocity was used as a computational approach to investigate potential transcriptional priming of the PGCLC expression program in d2 EpiLC. The RNA velocity analysis, which takes into account unspliced transcript reads for lineage tracing, did not show any evidence of transcriptional priming of d2 EpiLC (red) towards the PGCLC cluster (gray). In contrast, this same analysis revealed that a fraction of the cells profiled for the d2 EB (with a mixed mesodermal/PGCLC identity; gray arrow) seem to acquire a transcriptional program similar to the one found for PGCLC (shadowed in gray), indicating the feasibility of the RNA velocity method to detect transcriptional priming.

g.) Scatter plots comparing the transcriptomes of WT d2 EpiLC (x-axis) with those of d1.5 EpiLC, d3 EpiLC, *Prdm14*$^{-/-}$ d2 EpiLC or *Otx2*$^{-/-}$ d2 EpiLC (y-axis). All genes considered as expressed are shown as dots, with the PGCLC genes highlighted in green. The gene expression values are r-log normalized. The RNA-seq data was obtained from Yang *et al*. 2019[3], Buecker *et al*. 2014[4] and Shirane *et al*. 2016[5].

# Supplementary Fig. 2

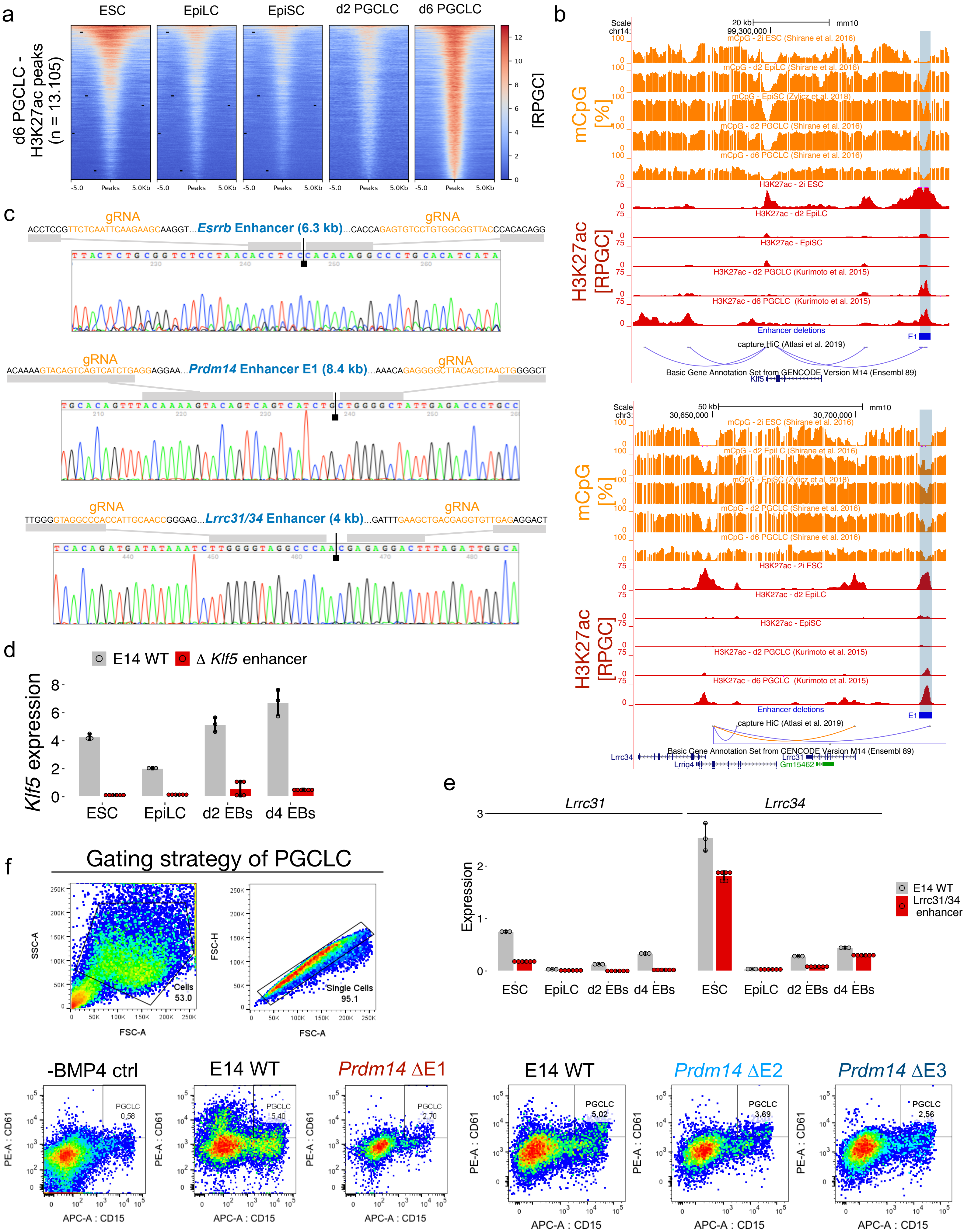

**Supplementary Fig. 2: Deletion and functional evaluation of representative PGCLC enhancers (related to Fig. 2).**

a.) Heatmaps showing H3K27ac ChIP-seq signals during PGCLC differentiation around all the distal H3K27ac peaks called in d6 PGCLC. The H3K27ac peaks were called using two H3K27ac ChIP-seq replicates generated in d6 PGCLC and only those peaks located >2 Kb from gene TSS were considered (see Methods). The H3K27ac ChIP-seq data from d2 and d6 PGCLC was obtained from Kurimoto *et al*. 2015[6]. RPGC: reads per genomic content.

b.) Genome-browser view of the H3K27ac and CpG methylation dynamics during PGCLC differentiation for the PGCLC enhancers, highlighted in blue, found within within the *Klf5* (top) and *Lrrc31/Lrrc34* (bottom) loci. The blue rectangles denote the enhancer deletions generated in mESC using CRISPR/Cas9 technology. According to capture Hi-C data generated in ESC[7], the *Klf5* enhancer is physically linked to the *Klf5* TSS, while the *Lrrc31/Lrrc34* enhancer is directly linked to the *Lrrc34* TSS, which itself interacts with *Lrrc31* (orange line). The H3K27ac PGCLC data was obtained from Kurimoto *et al.* 2015[6] and the CpG methylation data from Shirane *et al.* 2016[5]. RPGC: reads per genomic content.

c.) The different PGCLC enhancer deletions generated in ESC were confirmed by PCR genotyping followed by Sanger sequencing. This is illustrated by chromatograms showing the deletions generated for the *Esrrb*, *Prdm14* E1 and *Lrrc31/34* PGCLC enhancers. The reference sequence for each enhancer together with the corresponding CRISPR/Cas9 gRNAs (highlighted in orange) are shown above the chromatograms.

d-e.) *Klf5* (d)*, Lrrc31* and *Lrrc34* (e) expression were measured by RT-qPCR in d4 EB differentiated from WT ESC and two different ESC clonal lines with the *Lrrc31/Lrrc34* (d) or *Klf5* (e) enhancer deletions. The expression values were normalized to two housekeeping genes (*Eef1a1* and *Hprt*). The bar plots show the mean expression +/- SD from 6 measurements shown as circles (two clonal lines x three technical replicates).

f.) FACS gating strategy and representative examples of the PGCLC quantifications performed by FACS after four days of PGCLC differentiation using WT ESC (E14 WT) or ESC with deletions of each of the three *Prdm14* enhancers (ΔE1-E3). As a negative control, the WT ESC were also differentiated in the absence of BMP4 (-BMP4 ctrl). PGCLC were defined as the $CD15^+CD61^+$ cells found within d4 EB.

# Supplementary Fig. 3

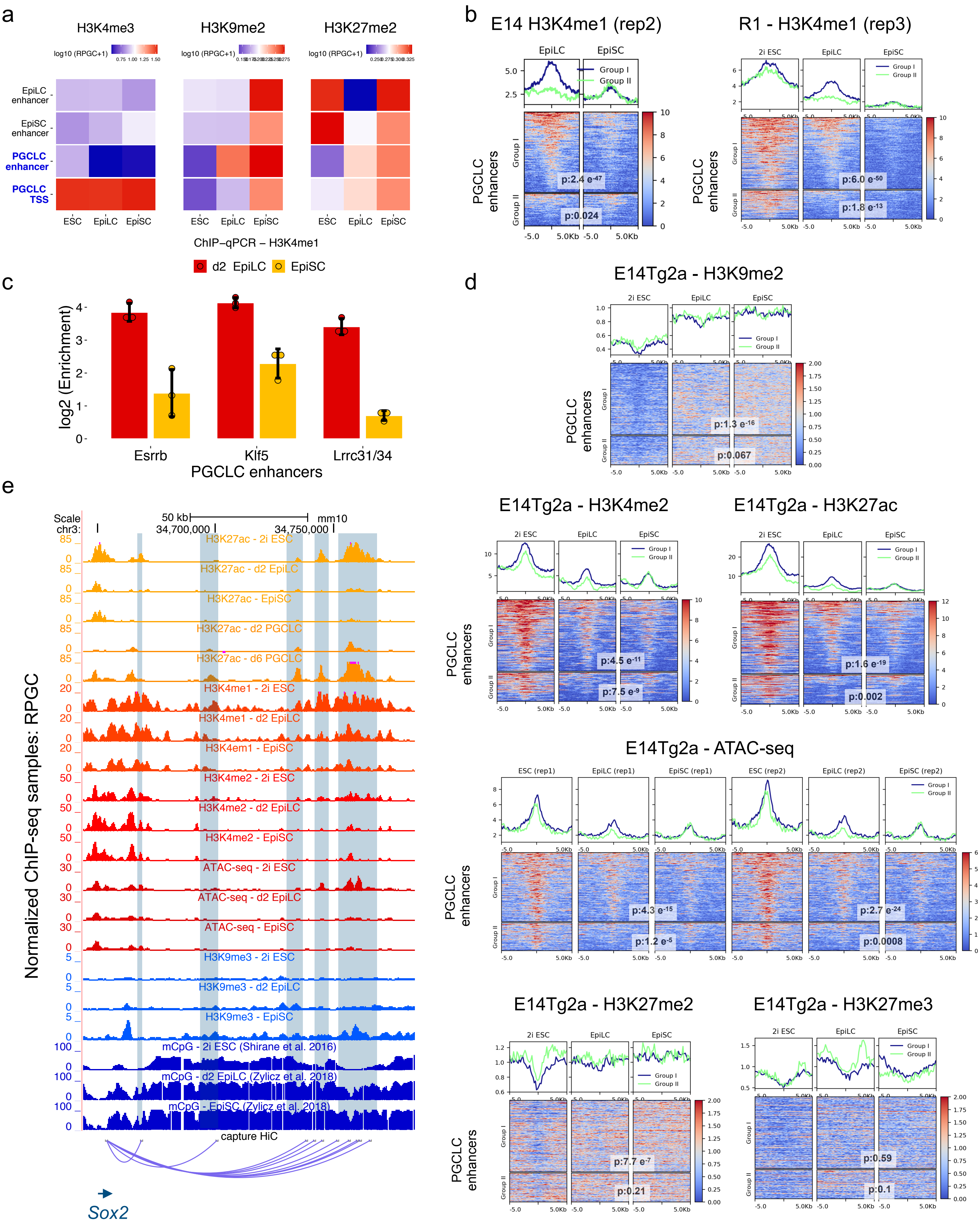

**Supplementary Fig. 3: Epigenomic profiling of PGCLC enhancers (related to Fig. 3).**

a.) Summary plot showing the average levels of H3K4me3, H3K9me2 and H3K27me2 measured in 2i ESC, d2 EpiLC and EpiSC for the EpiLC, EpiSC and PGCLC enhancers as well as the transcription start site (TSS) of the PGCLC genes. Quantifications were performed by measuring the average signals of each epigenetic mark within -/+ 1kb of the enhancers or TSS. RPGC: Reads per genomic content.

b.) Average profile (top) and heatmap (bottom) plots showing H3K4me1 levels for the Group I and Group II PGCLC enhancers in 2i ESC, d2 EpiLC and EpiSC. For the comparison between EpiLC and EpiSC, a paired Wilcoxon test was performed and the p-values for Group I and II PGCLC enhancers are indicated. The H3K4me1 signals correspond to the second (E14 cells) and third (R1 cells) ChIP-seq replicates generated in each cell type. Scales are shown in RPGC.

c.) H3K4me1 ChIP-qPCR analyses for the PGCLC enhancers of *Esrrb*, *Klf5* and *Lrrc31/34* in d2 EpiLC and EpiSC differentiated from E14 ESC. The H3K4me1 enrichments levels were normalized to a negative control region and are shown in log2 scale. The bar plots show the mean +/- SD from three technical replicates (circles).

d.) Average profile (top) and heatmap (bottom) plots showing H3K9me2, H3K4me2, H3K27ac and ATAC-seq (from two biological replicates), H3K27me2 and H3K27me3 levels for the Group I and Group II PGCLC enhancers in 2i ESC, d2 EpiLC and EpiSC. For the comparison between EpiLC and EpiSC, a paired Wilcoxon test was performed and the p-values for Group I and II PGCLC enhancers are indicated. Scales are shown in RPGC.

e.) Genome-browser view showing representative PGCLC enhancers interacting with *Sox2* according to capture Hi-C generated in ESC (Atlasi *et al.* 2019[7], blue arc) as well as the levels of several epigenetic marks in 2i ESC, d2 EpiLC and EpiSC. The H3K27ac PGCLC data was obtained from Kurimoto *et al.* 2015[6] and the CpG methylation data from Shirane *et al.* 2016[5] and Zylicz *et al.* 2015[8]. RPGC: reads per genomic content.

# Supplementary Fig. 4

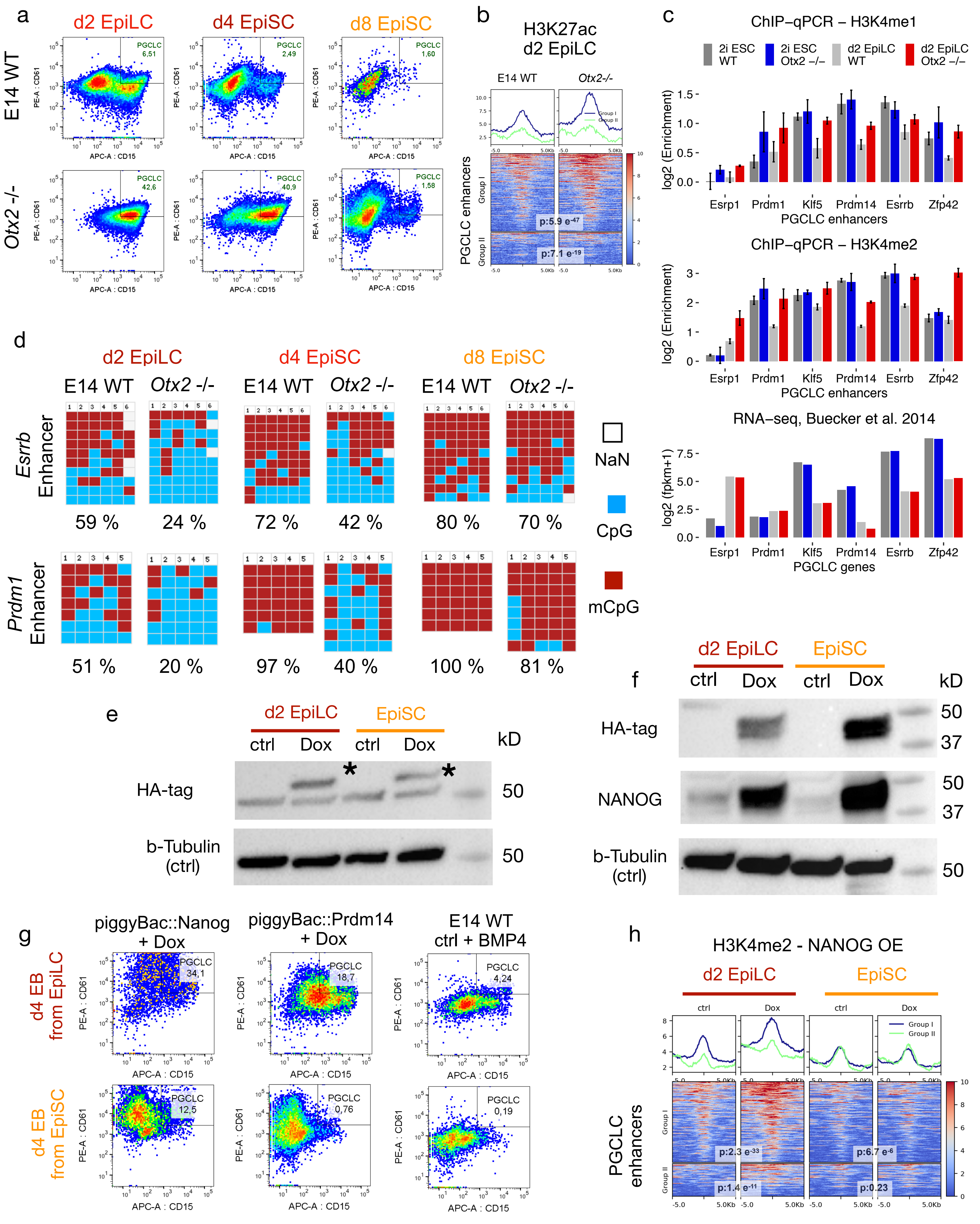

**Supplementary Fig. 4: Characterization of PGCLC enhancers in *Otx2*$^{-/-}$ cells and upon overexpression of major germline regulators (related to Fig. 4).**

a.) Representative examples of the PGCLC quantifications performed by FACS after four days of PGCLC differentiation starting from d2 EpiLC, d4 EpiSC and d8 EpiSC for either E14 WT (top) or E14 *Otx2*$^{-/-}$ (bottom) cells. PGCLC were quantified as the CD15$^{+}$CD61$^{+}$ cells found within d4 EB.

b.) Average profile (top) and heatmap (bottom) plots showing H3K27ac signals for the Group I and Group II PGCLC enhancers in E14 WT and *Otx2*$^{-/-}$ d2 EpiLC. For the comparison between WT and *Otx2*$^{-/-}$ EpiLC, a paired Wilcoxon test was performed and the p-values for Group I and II PGCLC enhancers are indicated. Scales are shown in RPGC.

c.) H3K4me1 (top) and H3K4me2 (middle) ChIP-qPCR analyses for selected PGCLC enhancers in E14 WT and *Otx2*$^{-/-}$ cells (2i ESC and d2 EpiLC). H3K4me1 and H3K4me2 enrichment levels were normalized to a negative control region and shown in log2 scale. The bar plots show the mean +/- SD from three technical replicates. The bottom panel shows the expression values for the genes associated with the selected PGCLC enhancers using RNA-seq data from Buecker *et al.* 2014[4].

d.) CpG methylation levels of selected PGCLC enhancers linked to *Esrrb* and *Prdm1* genes were measured by bisulfite sequencing in d2 EpiLC, d4 EpiSC and d8 EpiSC differentiated from E14 WT and *Otx2*$^{-/-}$ ESC. The columns of the plots correspond to individual CpG dinucleotides located within the indicated enhancer. Unmethylated CpGs are shown in blue, methylated CpGs in red and CpGs that were not sequenced are shown in gray. The rows represent the sequenced alleles in each cell line.

e-f.) Western blot analysis of the inducible overexpression of exogenous PRDM14-HA (e) and NANOG-HA (f). EpiLC or EpiSC were either left untreated (ctrl) or treated with Dox for 18 hours. (e) PRDM14-HA levels were measured using an anti-HA antibody and the band corresponding to PRDM14-HA is indicated with an asterisk. (f) NANOG levels were measured using anti-HA or anti-NANOG antibodies. B-Tubulin was used as a loading control. Western blots were performed once, but inducibility of PRDM14-HA and NANOG-HA was also confirmed by strong GFP expression upon Dox treatment, since the used piggybac vectors co-express the desired genes together with GFP due to an intervening IRES sequence. Source data is provided in the Source Data file.

g.) ESC lines enabling the inducible overexpression of exogenous NANOG-HA (piggyBac::*Nanog*) or PRMD14-HA (piggyBac::*Prdm14*) were differentiated into d2 EpiLC and EpiSC. Next, the resulting d2 EpiLC or EpiSC were differentiated into PGCLC upon overexpression (+Dox) of NANOG-HA or PRDM14-HA and in the absence of growth factors. Similarly, E14 WT d2 EpiLC and EpiSC were also differentiated into PGCLC in the presence of growth factors (ctrl + BMP4). Representative examples of the PGCLC quantifications performed by FACS after four days of PGCLC differentiation are shown. PGCLC were quantified as the percentage of CD15+ CD61+ cells found within d4 EB.

h.) The ESC line enabling the inducible overexpression of exogenous NANOG-HA was differentiated into d2 EpiLC and EpiSC. Next, the cells either left untreated (ctrl) or treated with Dox for 18 hours and H3K4me2 ChIP-seq experiments were performed. The average profile (top) and heatmap (bottom) plots show H3K4me2 levels within Group I and Group II PGCLC enhancers. For the comparison between the untreated (ctrl) and Dox-treated cells, a paired Wilcoxon test was performed and the p-values for Group I and II PGCLC enhancers are indicated. Scales are shown in RPGC.

# Supplementary Fig. 5

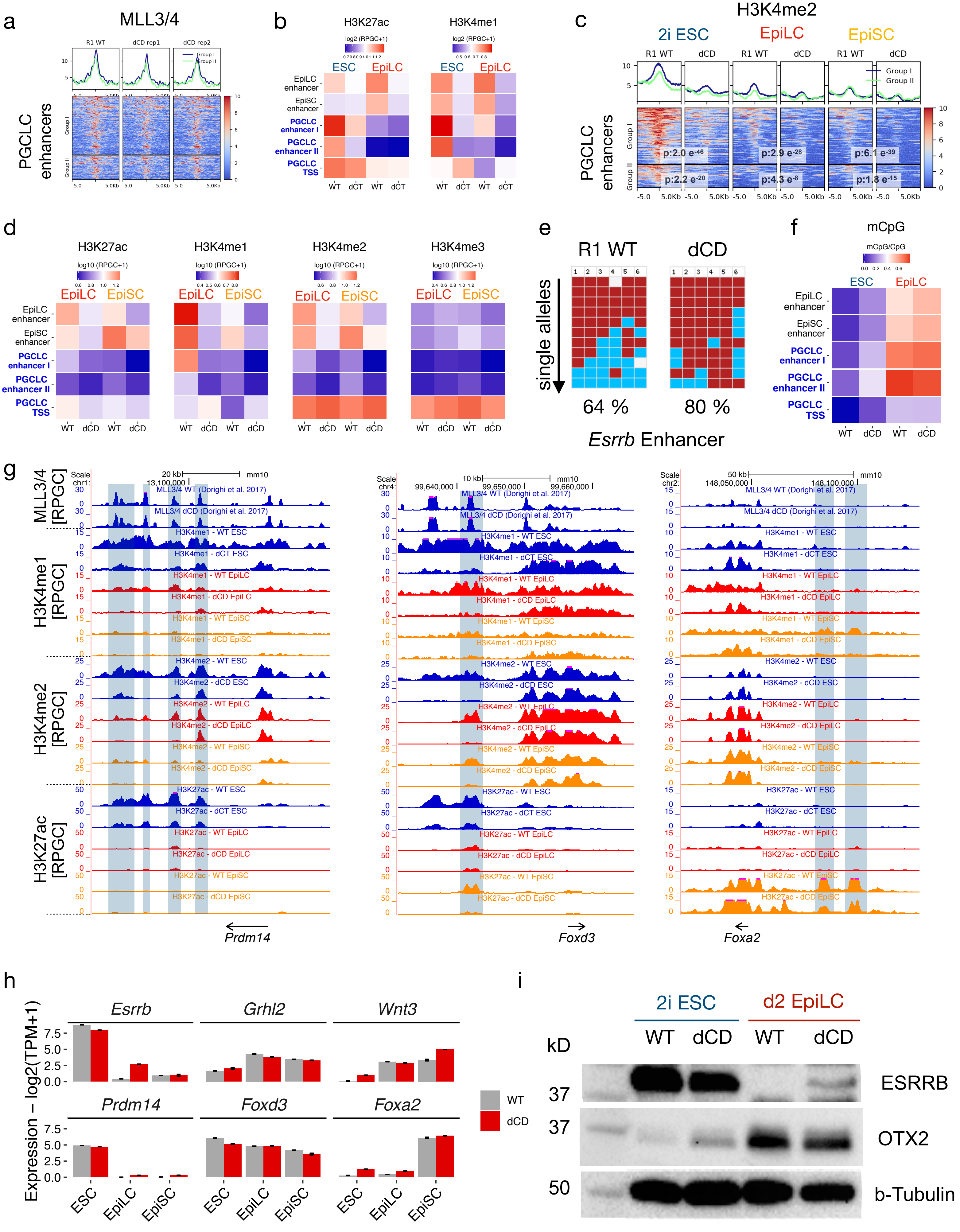

**Supplementary Fig. 5: Epigenomic and transcriptional characterization of MLL3/4 catalytic mutant cells (related to Fig. 5).**

a.) Average profile (top) and heatmap (bottom) plots showing the binding of WT and dCD MLL3/4 proteins for the Group I and II PGCLC enhancers in 2i ESC (ChIP-seq data from Dorighi *et al*. 2017[9]). The WT MLL3/4 ChIP-seq experiments were merged from two replicates. Scales are shown in RPGC.

b.) Summary plot showing the average H3K27ac and H3K4me1 levels in WT and dCT cells (ESC and d2 EpiLC) for the EpiLC, EpiSC and PGCLC enhancers as well as the TSS of the PGCLC genes. Quantifications were performed by measuring the average signals of each epigenetic mark within -/+ 1kb of the enhancers/TSS. RPGC: Reads per genomic content.

c.) Average profile (top) and heatmap (bottom) plots showing H3K4me2 levels for the Group I and Group II PGCLC enhancers in R1 WT and dCD ESC lines as well as upon their differentiation into d2 EpiLC and EpiSC. For the comparison between WT and dCD cells, a paired Wilcoxon test was performed for each stage and the p-values for Group I and II PGCLC enhancers are indicated. Scales are shown in RPGC.

d.) Summary plot showing the average H3K27ac, H3K4me1, H3K4me2 and H3K4me3 levels in WT and dCD cells (d2 EpiLC and EpiSC) for the EpiLC, EpiSC and PGCLC enhancers as well as the TSS of the PGCLC genes. Quantifications were performed by measuring the average signals of each epigenetic mark within -/+ 1kb of the enhancers or TSS. The H3K4me1 ChIP-seq data shown for WT d2 EpiLC and EpiSC are the same ones used in Fig. 3c as a third replicate. RPGC: Reads per genomic content.

e.) The CpG methylation levels of the *Esrrb* enhancer were determined by bisulfite sequencing in R1 WT and dCD d2 EpiLC. The columns of the plots correspond to individual CpG dinucleotides located within the *Esrrb* enhancer. Unmethylated CpGs are shown in blue, methylated CpGs in red and CpGs that were not sequenced are shown in gray. The rows represent the sequenced alleles in each cell line.

f.) Summary plot showing the average ratio of methylated CpG in WT and dCD cells (2i ESC and d2 EpiLC) for the EpiLC, EpiSC and PGCLC enhancers as well as the TSS of the PGCLC genes. Quantifications were performed by measuring the average signals of each epigenetic mark within -/ + 1kb of the enhancers or TSS.

g.) Genome browser view showing H3K4me1, H3K4me2 and H3K27ac profiles in both WT and MLL3/4 catalytic mutant cells (ESC, EpiLC and EpiSC) around representative PGCLC (*Prdm14)*, EpiLC (*Foxd3*) and EpiSC (*Foxa2*) genes and their associated enhancers.

h.) Expression levels, as measured by RNA-seq, for the representative PGCLC, EpiLC and EpiSC genes shown in Fig. 5c and Supplementary Fig. 5g in WT (gray) and dCD (red) ESC cells as well as upon their differentiation into EpiLC and EpiSC. The RNA-seq data in ESC was obtained from Dorighi *et al.* 2017[9] (n=3) and the experiments in d2 EpiLC and EpiSC were performed in duplicates. The barplots show the mean gene expression and the error bars represent the standard deviation from all available replicates TPM: transcripts per million.

i.) Western Blot analysis of the ESRRB and OTX2 protein levels present in WT and dCD ESC as well as upon their differentiation into d2 EpiLC. B-Tubulin was used as a loading control. Western blots were performed once, but similar results were observed in the RNA-seq data set. Source data provided in the Source Data file.

# Supplementary Fig. 6

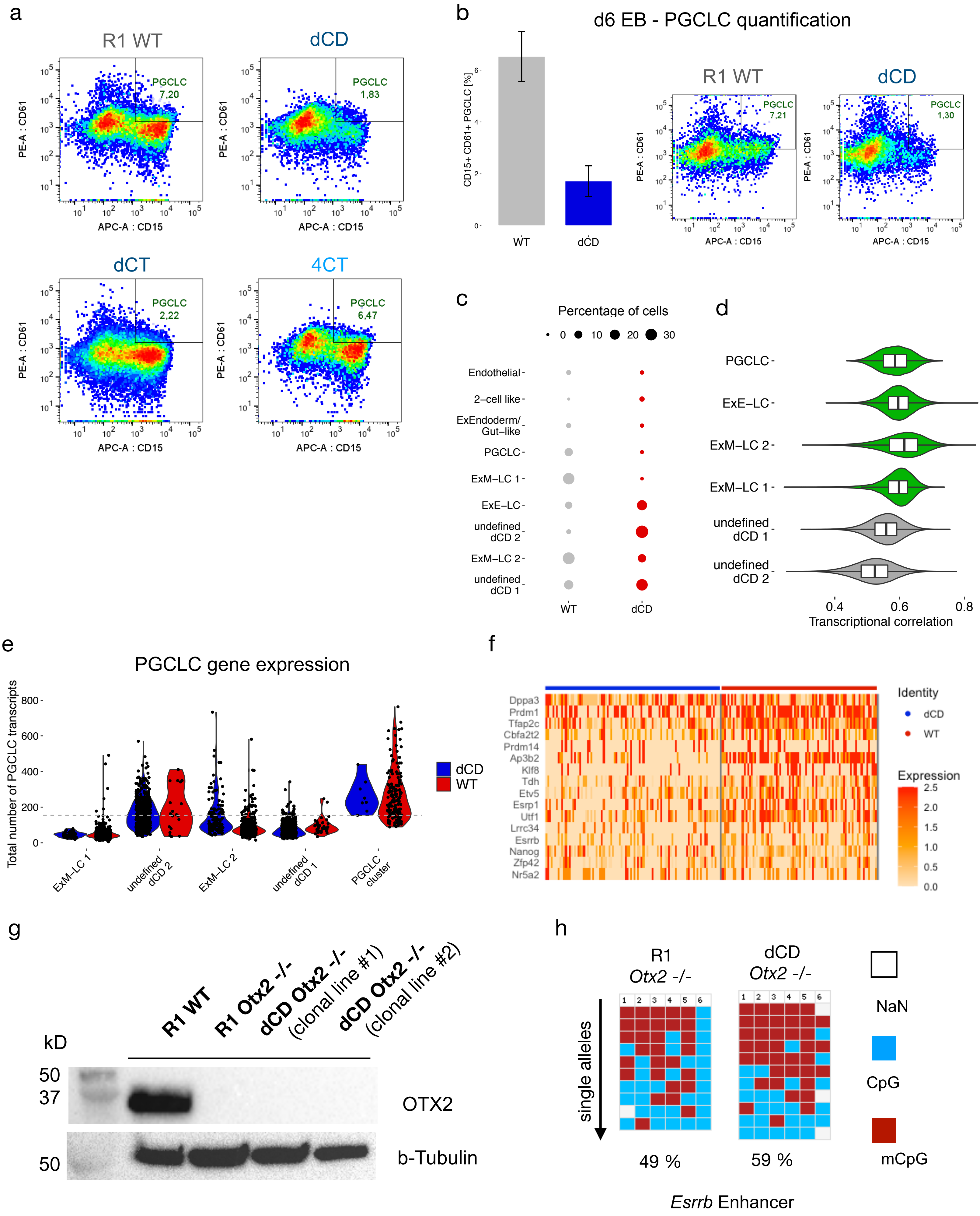

**Supplementary Fig. 6: Evaluation of the importance of H3K4me1 during PGCLC differentiation (related to Fig. 6).**

a.) Representative examples of the PGCLC quantifications performed by FACS after four days of PGCLC differentiation using WT ESC (R1 WT) or with the indicated MLL3/4 catalytic mutant ESC lines. PGCLC were quantified as the $CD15^+$ $CD61^+$ cells found within d4 EB.

b.) WT ESC and *Mll3/4* dCD ESC lines were differentiated into PGCLC and PGCLC were quantified in d6 instead of d4 EB. Examples are shown on the left and the PGCLC quantification of $CD15^+$ $CD61^+$ cells within d6 EB. The bar plots show the mean percentage of PGCLC +/- SD from two biological replicates.

c.) Percentage of WT and dCD cells present within each of the clusters identified in d4 EB as described in Fig. 6b.

d.) Transcriptional correlation of the main d4 EB clusters as described in (c-d). The violin plot shows the spearman correlations of the 1000 most variable genes between single cells of the indicated clusters. The horizontal lines in the box plots indicate the median, the boxes the first and third quartiles and the whiskers the $\pm 1.5 \times$ interquartile range.

e.) Violin plots showing the transcript levels of all PGCLC genes measured in either WT (red) or dCD (blue) cells located within the major cell clusters of the d4 EB clusters in WT (red) and dCD (blue). Each dot represents a single cell and the dashed lines show the mean transcript levels of the PGCLC genes for all the WT and dCD d4 EB cells except those found within the PGCLC cluster.

f.) Heatmap showing the expression of selected PGCLC genes (rows) linked to PGCLC enhancers within individual WT and dCD *Prdm1orDppa3+/Klf4-* cells (columns).

g.) Western blot analysis of the OTX2 levels in *Otx2*$^{-/-}$ and dCD *Otx2*$^{-/-}$ d2 EpiLC. B-Tubulin was used as a loading control. Western blots were performed once, but the *Otx2* deletion was also verified by genotyping and qPCR (not shown). Source data provided in the Source Data file.

h.) The CpG methylation levels of the *Esrrb* enhancer were determined by bisulfite sequencing in R1 *Otx2*$^{-/-}$ and dCD *Otx2*$^{-/-}$ d2 EpilC. The columns of the plots correspond to individual CpG dinucleotides located within the *Esrrb* enhancer. Unmethylated CpGs are shown in blue, methylated CpGs in red and CpGs that were not sequenced are shown in gray. The rows represent the sequenced alleles in each cell line.

# Supplementary Fig. 7

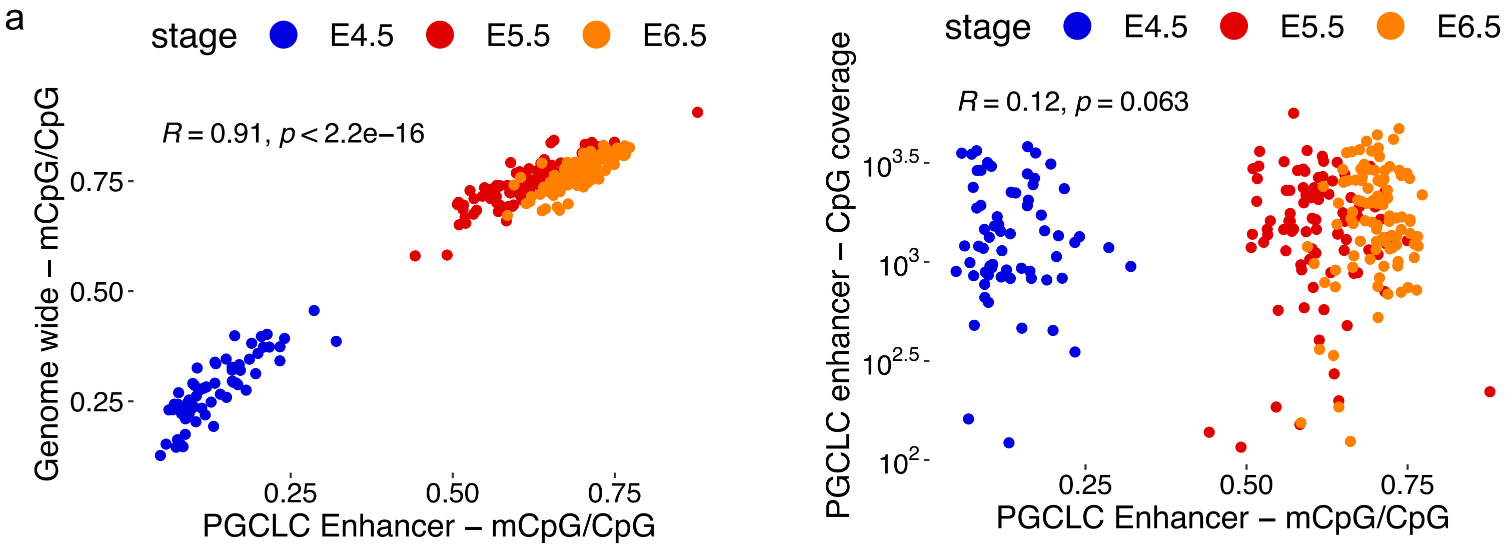


**Supplementary Fig. 7: Analysis of PGCLC enhancers using *in vivo* single-cell CpG methylation data (related to Fig. 7).**

a.) Scatterplots showing the correlation between (left) genome-wide mCpG or (right) CpG coverage and the mean mCpG/CpG ratio of all PGCLC enhancers within individual cells belonging to E4.5, E5.5 and E6.5 epiblasts (n=261 cells). The correlation coefficient (R) and p-value were calculated using Spearman correlation.

## References:

1: Ibarra-Soria, X. *et al.* Defining murine organogenesis at single-cell resolution reveals a role for the leukotriene pathway in regulating blood progenitor formation. *Nat. Cell Biol.* **20**, 127–134 (2018).

2: Nakaki, F. *et al.* Induction of mouse germ-cell fate by transcription factors in vitro. *Nature* **501**, 222–226 (2013).

3: Yang, P. *et al.* Multi-omic Profiling Reveals Dynamics of the Phased Progression of Pluripotency. *Cell Syst* **8**, 427–445.e10 (2019).

4: Buecker, C. *et al.* Reorganization of enhancer patterns in transition from naive to primed pluripotency. *Cell Stem Cell* **14**, 838–853 (2014).

5: Shirane, K. *et al.* Global Landscape and Regulatory Principles of DNA Methylation Reprogramming for Germ Cell Specification by Mouse Pluripotent Stem Cells. *Dev. Cell* **39**, 87–103 (2016).

6: Kurimoto, K. *et al.* Quantitative Dynamics of Chromatin Remodeling during Germ Cell Specification from Mouse Embryonic Stem Cells. *Cell Stem Cell* **16**, 517–532 (2015).

7: Atlasi, Y. *et al.* Epigenetic modulation of a hardwired 3D chromatin landscape in two naive states of pluripotency. *Nat. Cell Biol.* **21**, 568–578 (2019).

8: Zylicz, J. J. *et al.* Chromatin dynamics and the role of G9a in gene regulation and enhancer silencing during early mouse development. *Elife* **4**, (2015).

9: Dorighi, K. M. *et al.* Mll3 and Mll4 Facilitate Enhancer RNA Synthesis and Transcription from Promoters Independently of H3K4 Monomethylation. *Mol. Cell* **66**, 568–576.e4 (2017).
