# Supplementary material for: Enhancer-associated H3K4 methylation safeguards in vitro germline competence
Source: Nat Commun. 2021 Oct 1;12:5771. doi: 10.1038/s41467-021-26065-6 (PMC8486853; doi:10.1038/s41467-021-26065-6)
Supplement: Supplementary file 3 — Description of Additional Supplementary Files [file 41467_2021_26065_MOESM3_ESM.pdf]

Description of Additional Supplementary Files

File Name: Supplementary Data 1

Description: UMI count matrix of all cells analyzed across different stages of PGCLC differentiation.

File Name: Supplementary Data 2

Description: ESC, EpiLC, EpiSC and PGCLC gene sets based on single-cell RNA-seq profiling across different stages of PGCLC differentiation.

File Name: Supplementary Data 3

Description: EpiLC, EpiSC and PGCLC enhancers coordinates and linked genes.

File Name: Supplementary Data 4

Description: RNA-seq results in WT vs Mll3/4 dCD cells.

File Name: Supplementary Data 5

Description: UMI count matrix of all cells analyzed from d4 EB (WT & dCD).

File Name: Supplementary Data 6

Description: Resources
