# Supplementary material for: Enhancer-associated H3K4 methylation safeguards in vitro germline competence
Source: Nat Commun. 2021 Oct 1;12:5771. doi: 10.1038/s41467-021-26065-6 (PMC8486853; doi:10.1038/s41467-021-26065-6)
Supplement: Supplementary file 10 — Reporting Summary [file 41467_2021_26065_MOESM10_ESM.pdf]

nature portfolio

Corresponding author(s): Alvaro Rada-Iglesias

Last updated by author(s): Sep 1, 2021

# Reporting Summary

Nature Portfolio wishes to improve the reproducibility of the work that we publish. This form provides structure for consistency and transparency in reporting. For further information on Nature Portfolio policies, see our Editorial Policies and the Editorial Policy Checklist.

## Statistics

For all statistical analyses, confirm that the following items are present in the figure legend, table legend, main text, or Methods section.

| n/a | Confirmed | |
|---|---|---|
| ☐ | ☒ | The exact sample size ($n$) for each experimental group/condition, given as a discrete number and unit of measurement |
| ☐ | ☒ | A statement on whether measurements were taken from distinct samples or whether the same sample was measured repeatedly |
| ☐ | ☒ | The statistical test(s) used AND whether they are one- or two-sided<br>*Only common tests should be described solely by name; describe more complex techniques in the Methods section.* |
| ☒ | ☐ | A description of all covariates tested |
| ☒ | ☐ | A description of any assumptions or corrections, such as tests of normality and adjustment for multiple comparisons |
| ☐ | ☒ | A full description of the statistical parameters including central tendency (e.g. means) or other basic estimates (e.g. regression coefficient) AND variation (e.g. standard deviation) or associated estimates of uncertainty (e.g. confidence intervals) |
| ☐ | ☒ | For null hypothesis testing, the test statistic (e.g. $F$, $t$, $r$) with confidence intervals, effect sizes, degrees of freedom and $P$ value noted<br>*Give P values as exact values whenever suitable.* |
| ☒ | ☐ | For Bayesian analysis, information on the choice of priors and Markov chain Monte Carlo settings |
| ☒ | ☐ | For hierarchical and complex designs, identification of the appropriate level for tests and full reporting of outcomes |
| ☐ | ☒ | Estimates of effect sizes (e.g. Cohen's $d$, Pearson's $r$), indicating how they were calculated |

*Our web collection on statistics for biologists contains articles on many of the points above.*

## Software and code

Policy information about availability of computer code

| | |
|---|---|
| Data collection | RT-qPCR: Light Cycler 480II<br>FACS: FACS Canto II Cytometer / FACS AriaIII cell sorter with BD FACSDiva Software (version 5.0.3)<br>Next-generation sequencing: NovaSeq 6000 System (Illumina) |
| Data analysis | All analysis code is available at Github: https://github.com/ToreBle/Germline_competence<br>Furthermore the following programmes and R packages were used for the data analysis:<br>AnnotationHub 2.18.0<br>bedtools 2.29.2<br>BISMA<br>Bismark v0.16.1<br>bowtie2 2.2.9<br>BUSpaRse 1.0.0<br>bwa 0.7.15<br>cellranger 2.1.0<br>cutadapt 2.8<br>deeptools 3.3.1<br>DESeq2 1.26.0<br>edgeR 3.28.1<br>FlowJo 10.6.1<br>GREAT 4.0.4<br>kallisto 0.46.2<br>Loupe Browser 4.2.0 |

macs2 2.2.6
monocle 2.14.0
novoalignCS V1.06.09
picard 2.5.0
R 3.6.1
Seurat 3.9.9.9035
STAR 2.2.1
velocyto.R 0.6
samtools 1.3.1
Trim Galore 0.4.2
UCSC Genome Browser

For manuscripts utilizing custom algorithms or software that are central to the research but not yet described in published literature, software must be made available to editors and reviewers. We strongly encourage code deposition in a community repository (e.g. GitHub). See the Nature Portfolio guidelines for submitting code & software for further information.

## Data

Policy information about availability of data

All manuscripts must include a data availability statement. This statement should provide the following information, where applicable:

- Accession codes, unique identifiers, or web links for publicly available datasets
- A description of any restrictions on data availability
- For clinical datasets or third party data, please ensure that the statement adheres to our policy

The sequencing datasets generated during this study are available through GEO (GSE155089): https://www.ncbi.nlm.nih.gov/geo/query/acc.cgi?acc=GSE155089

All data are he different types of genomic data (i.e. ChIP-seq, ATAC-seq, genome-wide bisulftie sequencing (WGBS), 4C-seq, RNA-seq and single-cell RNA-seq) can be directly accessed using the follwoing GEO accession numbers:
GSE155015 4C-seq
GSE155058 ATAC-seq
GSE155062 ChIP-seq 1
GSE155069 ChIP-seq 2
GSE155079 RNA-seq
GSE155083 WGBS
GSE155088 scRNA-seq

# Field-specific reporting

Please select the one below that is the best fit for your research. If you are not sure, read the appropriate sections before making your selection.

☒ Life sciences ☐ Behavioural & social sciences ☐ Ecological, evolutionary & environmental sciences

For a reference copy of the document with all sections, see nature.com/documents/nr-reporting-summary-flat.pdf

# Life sciences study design

All studies must disclose on these points even when the disclosure is negative.

| | |
|---|---|
| Sample size | No statistical methods were used to predetermine sample size. For the transgenic ESC lines generated for this study, at least two clonal lines with the same genotype were analyzed. As this resulted in similar results, we decided to keep a minimum of two clonal lines for all investigated transgenic ESC. |
| Data exclusions | No data was excluded. |
| Replication | All experiments were performed independently at least twice and all the attempts at data replication were successful. The exact number of repicates is described in the corresponding figure legends. |
| Randomization | Randomization was not relevant for our study as the sample sizes of the different experiments were too small for randomization. |
| Blinding | The investigators were not blinded to allocation during experiments and outcome assessment. There was one person in charge of generating and characterizing all cell lines and performing bioinformatical analysis. Blinding is typically used with randomization and large sample sizes, which, as stated before, does not apply to our experiments. |

# Reporting for specific materials, systems and methods

We require information from authors about some types of materials, experimental systems and methods used in many studies. Here, indicate whether each material, system or method listed is relevant to your study. If you are not sure if a list item applies to your research, read the appropriate section before selecting a response.

## Materials & experimental systems

| n/a | Involved in the study | |
|---|---|---|
| ☐ | ☒ | Antibodies |
| ☐ | ☒ | Eukaryotic cell lines |
| ☒ | ☐ | Palaeontology and archaeology |
| ☒ | ☐ | Animals and other organisms |
| ☒ | ☐ | Human research participants |
| ☒ | ☐ | Clinical data |
| ☒ | ☐ | Dual use research of concern |

## Methods

| n/a | Involved in the study | |
|---|---|---|
| ☐ | ☒ | ChIP-seq |
| ☐ | ☒ | Flow cytometry |
| ☒ | ☐ | MRI-based neuroimaging |

# Antibodies

Antibodies used

Antibody Source ID Application Dilution
H3K4me1 Active Motif 39297 ChIP-seq 4 µl
H3K4me2 Active Motif 39141 ChIP-seq 4 µl
H3K4me3 Active Motif 39159 ChIP-seq 4 µl
H3K9me2 Active Motif 39683 ChIP-seq 5 µl
H3K9me3 Active Motif 39161 ChIP-seq 5 µl
H3K27ac Active Motif 39133 ChIP-seq 4 µl
H3K27me2 Cell Signaling 9728S ChIP-seq 5 µl
H3K27me3 Active Motif 39155 ChIP-seq 5 µl
HA Abcam ab9110 ChIP-seq 4 µl

HA-tag Abcam ab9110 Western Blot 1:5000
NANOG Bethyl A300-397A Western Blot 1:2000
ESRRB Perseus Proteomics PP-H6705-00 Western Blot 1:1000
OTX2 Proteintech 13497-1-AP Western Blot 1:1500
B-TUBULIN Sigma-Aldrich T0198 Western Blot 1:2000

CD15 Biolegend 104307 FACS 1:500
CD61 Thermo Fisher Scientific 50-8813-41 FACS 1:200

Validation

H3K4me1: https://www.activemotif.com/catalog/details/39297/histone-h3-monomethyl-lys4-antibody-pab
H3K4me2: https://www.activemotif.com/catalog/details/39141/histone-h3-dimethyl-lys4-antibody-pab
H3K4me3: https://www.activemotif.com/catalog/details/39159/histone-h3-trimethyl-lys4-antibody-pab
H3K9me2: https://www.activemotif.com/catalog/details/39683/histone-h3-dimethyl-lys9-antibody-mab-clone-mabi-0307
H3K9me3: https://www.activemotif.com/catalog/details/39161/histone-h3-trimethyl-lys9-antibody-pab
H3K27ac: https://www.activemotif.com/catalog/details/39133/histone-h3-acetyl-lys27-antibody-pab
H3K27me2: https://www.cellsignal.com/products/primary-antibodies/di-methyl-histone-h3-lys27-d18c8-xp-rabbit-mab/9728?N=4294956287&Ntt=9728S&fromPage=plp
H3K27me3: https://www.activemotif.com/catalog/details/39155/histone-h3-trimethyl-lys27-antibody-pab
HA: https://www.abcam.com/ha-tag-antibody-chip-grade-ab9110.html

NANOG: https://www.bethyl.com/product/A300-397A/Nanog_Antibody
ESRRB: https://www.bio-techne.com/p/antibodies/human-err-beta-nr3b2-antibody-h6705_pp-h6705-00#technical-data-tab-tab
OTX2: https://www.ptglab.com/products/OTX2-Antibody-13497-1-AP.htm
B-TUBULIN: https://www.sigmaaldrich.com/DE/de/product/sigma/t0198
C15 and CD61: Hayashi and Saitou, 2031 - doi: 10.1038/nprot.2013.090

# Eukaryotic cell lines

Policy information about cell lines

Cell line source(s)

Two different male mouse ESC lines (i.e. E14Tg2a and R1) were used as indicated for each particular experiment.
All enhancer deletions (Prdm14 E1, Prdm14 E2, Prdm14 E3, Esrrb E1, Klf5 E1 and Lrrc31 E1) were generated in E14Tg2a using CRISPR/Cas9 and the gRNAs described in Supplementary Data 6. For each experiment two clonal lines have been analysed.
The Mll3/4 dCD, Mll3/4 dCT and Mll4 CT ESC lines were derived from WT R1 ESC as described in (Dorighi et al., 2017). The R1 Otx2 -/- and dCD Otx2 -/- cell line were generated with a full Otx2 KO using CRISPR/Cas9 in the R1 WT and Mll3/4 dCD cell line, respectively.
The E14 Otx2 -/- ESC line (Acampora et al., 2013; Buecker et al., 2014) and its parental E14Tg2a ESC were kindly provided by Christa Buecker.

Authentication

The enhancer and gene deletions were authenticated by PCR genotyping and Sanger-sequencing. The Otx2 deletions were validated additionally by western blot (Supplementary Fig. S6f) and the amino acid substitutions of Mll3 were confirmed by

| | |
|---|---|
| | Sanger-sequencing. The loss of H3K4me1/2 in dCD and dCT cells was confirmed by ChIP-seq and ChIP-qPCR. |
| Mycoplasma contamination | The WT E14 mESC have been previously tested; all the remaining ESC lines were not tested. |
| Commonly misidentified lines (See ICLAC register) | None |

# ChIP-seq

## Data deposition

☒ Confirm that both raw and final processed data have been deposited in a public database such as GEO.

☒ Confirm that you have deposited or provided access to graph files (e.g. BED files) for the called peaks.

| | |
|---|---|
| Data access links *May remain private before publication.* | https://www.ncbi.nlm.nih.gov/geo/query/acc.cgi?acc=GSE155089 |
| Files in database submission | GSM4693166 4C_Prdm14_TSS_ESC<br>GSM4693167 4C_Prdm14_TSS_EpiLC<br>GSM4693168 4C_Prdm14_TSS_EpiSC<br>GSM4694522 ATAC_input<br>GSM4694523 ATAC_ESC_rep1<br>GSM4694524 ATAC_ESC_rep2<br>GSM4694525 ATAC_EpiLC_rep1<br>GSM4694526 ATAC_EpiLC_rep2<br>GSM4694527 ATAC_EpiSC_rep1<br>GSM4694528 ATAC_EpiSC_rep2<br>GSM4694551 H3K4me1_ESC<br>GSM4694552 H3K4me1_EpiLC<br>GSM4694553 H3K4me1_EpiSC<br>GSM4694554 H3K9me2_ESC<br>GSM4694555 H3K9me2_EpiLC<br>GSM4694556 H3K9me2_EpiSC<br>GSM4694557 H3K9me3_ESC<br>GSM4694558 H3K9me3_EpiLC<br>GSM4694559 H3K9me3_EpiSC<br>GSM4694560 H3K27ac_ESC_WT<br>GSM4694561 H3K27ac_EpiLC_WT<br>GSM4694562 H3K27ac_EpiSC_WT<br>GSM4694563 H3K4me2_ESC<br>GSM4694564 H3K4me2_EpiLC<br>GSM4694565 H3K4me2_EpiSC<br>GSM4694566 H3K27me2_ESC_WT<br>GSM4694567 H3K27me2_EpiLC_WT<br>GSM4694568 H3K27me2_EpiSC_WT<br>GSM4694569 ChIP_input_ESC<br>GSM4694570 ChIP_input_EpiLC<br>GSM4694571 ChIP_input_EpiSC<br>GSM4694572 H3K4me3_ESC<br>GSM4694573 H3K4me3_EpiLC<br>GSM4694574 H3K4me3_EpiSC<br>GSM4694575 H3K27me3_ESC_WT<br>GSM4694576 H3K27me3_EpiLC_WT<br>GSM4694577 H3K27me3_EpiSC_WT<br>GSM4694578 HA_EpiLC_WT<br>GSM4694579 HA_EpiLC_Prdm14<br>GSM4694580 HA_EpiLC_Nanog<br>GSM4694581 HA_EpiSC_WT<br>GSM4694582 HA_EpiSC_Prdm14<br>GSM4694583 HA_EpiSC_Nanog_rep1<br>GSM4694584 HA_EpiSC_Nanog_rep2<br>GSM4694585 H3K27ac_EpiLC_ctrl<br>GSM4694586 H3K4me2_EpiLC_ctrl<br>GSM4694587 H3K27ac_EpiLC_Dox<br>GSM4694588 H3K4me2_EpiLC_Dox<br>GSM4694589 H3K27ac_EpiSC_ctrl<br>GSM4694590 H3K4me2_EpiSC_ctrl<br>GSM4694591 H3K27ac_EpiSC_Dox<br>GSM4694592 H3K4me2_EpiSC_Dox |

GSM4694701 ChIP_H3K4me1_EpiLC_WT_rep1
GSM4694702 ChIP_H3K4me1_EpiLC_dCD
GSM4694703 ChIP_H3K4me1_EpiSC_WT
GSM4694704 ChIP_H3K4me1_EpiSC_dCD
GSM4694705 ChIP_H3K27ac_EpiLC_WT_rep1
GSM4694706 ChIP_H3K27ac_EpiLC_dCD
GSM4694707 ChIP_H3K27ac_EpiSC_WT
GSM4694708 ChIP_H3K27ac_EpiSC_dCD
GSM4694709 ChIP_H3K4me1_ESC_WT
GSM4694710 ChIP_H3K4me1_ESC_dCT
GSM4694711 ChIP_H3K4me1_EpiLC_WT_rep2
GSM4694712 ChIP_H3K4me1_EpiLC_dCT
GSM4694713 ChIP_H3K27ac_ESC_WT
GSM4694714 ChIP_H3K27ac_ESC_dCT
GSM4694715 ChIP_H3K27ac_EpiLC_WT_rep2
GSM4694716 ChIP_H3K27ac_EpiLC_dCT
GSM4694717 ChIP_H3K27ac_d4EB_WT
GSM4694718 ChIP_H3K27ac_d4EB_dCD
GSM4694719 ChIP_H3K4me2_ESC_WT
GSM4694720 ChIP_H3K4me2_ESC_dCD
GSM4694721 ChIP_H3K4me2_EpiLC_WT
GSM4694722 ChIP_H3K4me2_EpiLC_dCD
GSM4694723 ChIP_H3K4me2_EpiSC_WT
GSM4694724 ChIP_H3K4me2_EpiSC_dCD
GSM4694725 ChIP_H3K27ac_d2EpiLC_WT
GSM4694726 ChIP_H3K4me1_d2EpiLC_WT
GSM4694727 ChIP_H3K4me2_d2EpiLC_WT
GSM4694728 ChIP_H3K27ac_d2EpiLC_Otx2
GSM4694729 ChIP_H3K4me1_d2EpiLC_Otx2
GSM4694730 ChIP_H3K4me2_d2EpiLC_Otx2
GSM4694731 ChIP_H3K4me1_d4EpiSC_WT
GSM4694732 ChIP_H3K4me2_d4EpiSC_WT
GSM4694733 ChIP_H3K4me1_d4EpiSC_Otx2
GSM4694734 ChIP_H3K4me2_d4EpiSC_Otx2
GSM4694735 ChIP_H3K4me1_d8EpiSC_WT
GSM4694736 ChIP_H3K4me2_d8EpiSC_WT
GSM4694737 ChIP_H3K4me1_d8EpiSC_Otx2
GSM4694738 ChIP_H3K4me2_d8EpiSC_Otx2
GSM4694849 RNA_EpiLC_WT_rep1
GSM4694850 RNA_EpiLC_WT_rep2
GSM4694851 RNA_EpILC_dCD_rep1
GSM4694852 RNA_EpILC_dCD_rep2
GSM4694853 RNA_EpiSC_WT_rep1
GSM4694854 RNA_EpiSC_WT_rep2
GSM4694855 RNA_EpiSC_dCD_rep1
GSM4694856 RNA_EpiSC_dCD_rep2
GSM4694889 PBAT_EpiLC_WT
GSM4694890 PBAT_EpiLC_Otx2
GSM4694891 PBAT_EpiLC_dCD
GSM4694892 PBAT_EpiLC_dCD_Otx2
GSM4694997 scRNAseq_2i_ESC
GSM4694998 scRNAseq_d2_EpiLC
GSM4694999 scRNAseq_d1_EpiLC
GSM4695000 scRNAseq_d2_EB
GSM4695001 scRNAseq_EpiSC
GSM4695002 scRNAseq_d4_EB
GSM4695003 scRNAseq_SL_ESC
GSM4695004 scRNAseq_d3_EpiLC
GSM5034698 scRNAseq_d4EB_WT
GSM5034699 scRNAseq_d4EB_dCD
GSM5034700 ChIP_H3K4me3_ESC_WT
GSM5034701 ChIP_H3K4me3_ESC_dCD
GSM5034702 ChIP_H3K4me3_EpiLC_WT
GSM5034703 ChIP_H3K4me3_EpiLC_dCD
GSM5034704 ChIP_H3K4me3_EpiSC_WT
GSM5034705 ChIP_H3K4me3_EpiSC_dCD

Genome browser session
(e.g. UCSC)

Comparison of the pluripotent stages (related to Fig. 2):
http://genome-euro.ucsc.edu/s/Tore/Comparison%20of%20the%20pluripotent%20stages

PRDM14/NANOG overexpression and topological features (related to Fig. 5):
http://genome-euro.ucsc.edu/cgi-bin/hgTracks?hgS_doOtherUser=submit&hgS_otherUserName=Tore&hgS_otherUserSessionName=PRDM14%2FNANOG%20overexpression

H3K4me1/2 deficiency and Otx2-/- (related to Fig. 6,7):
http://genome-euro.ucsc.edu/cgi-bin/hgTracks?hgS_doOtherUser=submit&hgS_otherUserName=Tore&hgS_otherUserSessionName=H3K4me1%2F2%20deficiency%20and%20Otx2%2D%2F%2D

## Methodology

**Replicates**

ATAC-seq experiments were performed in 2 replicates for each condition (ESC, EpiLC, EpiSC).

H3K4me1 ChIP-seq experiments in ESC (n = 2), EpiLC (n = 4) and EpiSC (n = 2) performed in R1 and E14Tg2a cell lines.
H3K4me2 ChIP-seq experiments in ESC (n = 2), EpiLC (n = 3) and EpiSC (n = 3) performed in R1 and E14Tg2a cell lines.
H3K4me3 ChIP-seq experiments in ESC (n = 2), EpiLC (n = 2) and EpiSC (n = 2) performed in R1 and E14Tg2a cell lines.
H3K27ac ChIP-seq experiments in ESC (n = 2), EpiLC (n = 4) and EpiSC (n=2) performed in R1 and E14Tg2a cell lines.
NANOG-HA ChIP-seq experiments in EpiSC were performed as two biological replicates in E14Tg2a.

RNA-seq experiments were performed in at least 2 replicates for each condition (EpiLC WT, EpiLC dCD, EpiSC WT, EpiSC dCD)

**Sequencing depth**

ChIP-seq experiments were performed with 1x50 bp with at least 30 million reads per sample.

**Antibodies**

Antibody Source  ID Application Dilution
H3K4me1 Active Motif 39297 ChIP-seq 4 µl
H3K4me2 Active Motif 39141 ChIP-seq 4 µl
H3K4me3 Active Motif 39159 ChIP-seq 4 µl
H3K9me2 Active Motif 39683 ChIP-seq 5 µl
H3K9me3 Active Motif 39161 ChIP-seq 5 µl
H3K27ac Active Motif 39133 ChIP-seq 4 µl
H3K27me2 Cell Signaling 9728S ChIP-seq 5 µl
H3K27me3 Active Motif 39155 ChIP-seq 5 µl
HA Abcam ab9110 ChIP-seq 4 µl

**Peak calling parameters**

Peaks were only called from H3K27ac ChIP-seq, including public H3K27ac ChIP-seq data of PGCLC (Kurimoto et al. 2016/ GEO accession: GSE60204).
MACS2 were used to call peaks with the following setting --broad -m 5 50 --fix-bimodal --extsize 200

**Data quality**

For the H3K27ac peaks in EpiLC, EpiSC and PGCLC only peaks with q-values < 1 x 10-3 were considered.

**Software**

Mapping (mm10): bwa mem aligner
De-duplication: Picard tools-2.9.0-1
Remove ENCODE blacklisted regions and big Wig files: deeptools-3.3.1
All analysis code is publically available at Github: https://github.com/ToreBle/Germline_competence

# Flow Cytometry

## Plots

Confirm that:

- [x] The axis labels state the marker and fluorochrome used (e.g. CD4-FITC).
- [x] The axis scales are clearly visible. Include numbers along axes only for bottom left plot of group (a 'group' is an analysis of identical markers).
- [x] All plots are contour plots with outliers or pseudocolor plots.
- [x] A numerical value for number of cells or percentage (with statistics) is provided.

## Methodology

**Sample preparation**

According to the protocol from Hayashi and Saitou, 2013, embryoid bodies (EB) containing primordial germ cell like cells (PGCLC) were dissociated and filtered into single cells using trypsin. Each measurement was performed at least for two biological replicates. More details are described in the methods.

**Instrument**

FACS Canto Cytometer (PGCLC quantification) and FACS AriaIII cell sorter (PGCLC sorting)

**Software**

Data collection: BD FACSDiva Software version 5.0.3
Data analysis: FlowJo 10.6.1

| | |
|---|---|
| Cell population abundance | As described previously (Hayashi and Saitou, 2013), about 5 - 10 % of PGCLC were obtained as CD15+ CD61+. |
| Gating strategy | Initially the cell populations were identified with FSC-A (Area)/SSC-A (Area) plots. A polygon gate was used to separate the cells from debris. Then, in a FSC-H (Height) / FSC-A (Area) plot, single cells were selected and doublets were excluded. The boundaries for the PGCLC quantification were defined by single stained controls or control EB cells which were differentiated in the absence of BMP4 and thus did not induce PGCLC. The gating strategy is provided in Supplementary Figure 2f. |

☒ Tick this box to confirm that a figure exemplifying the gating strategy is provided in the Supplementary Information.
